# Nitrogen cycling genes abundance in soil and aboveground compartments of tropical peatland cloud forests and a wetland on Réunion Island

**DOI:** 10.1038/s41598-025-12367-y

**Published:** 2025-07-25

**Authors:** Fahad Ali Kazmi, Ülo Mander, Reti Ranniku, Maarja Öpik, Kersti Püssa, Kaido Soosaar, Kuno Kasak, Mohit Masta, Claudine Ah-Peng, Mikk Espenberg

**Affiliations:** 1 Department of Geography, University of Tartu, 51003 Tartu, Estonia; 2 Department of Biological and Agricultural Engineering, University of Arkansas, Fayetteville, AR 72701 USA; 3 Department of Botany, University of Tartu, 50409 Tartu, Estonia; 4 Department of Environmental Science, Policy and Management, University of California, Berkeley, USA; 5 UMR PVBMT, Université de La Réunion, 97410 Saint-Pierre, La Réunion France; 6 OSU-Réunion, Université de La Réunion, 97400 Saint-Denis, La Réunion France

**Keywords:** Soil Microbiome, Canopy Microbiome, Soil N_2_O, Soil N_2_, Stem N_2_O, Microbiology, Biogeochemistry

## Abstract

Peatland cloud forests, characterized by high altitude and humidity, are among the least-studied tropical ecosystems despite their significance for endemism and the bioavailable nitrogen (N) that can be emitted as N_2_O. While research has mainly focused on soil, the above-ground microbial N cycle remains largely unexplored. We quantified microbial N cycling genes across ecosystem compartments (soil, canopy soil, tree stems, and leaves) in relation to N_2_O and N_2_ fluxes and soil physicochemical properties in two peatland cloud forests and a wetland on Réunion Island. Complete denitrification minimized N_2_O emissions and increased N_2_ fluxes in wetland soils. In cloud forest soils, archaeal nitrification primarily produced nitrate (NO_3_^–^), while low pH potentially slowed denitrification, resulting in minimal N_2_O emissions. Soil N-fixers were more abundant in *Erica reunionensis*-dominated forests than in mixed forests. Tree stems varied between weak N_2_O sinks and sources, with fluxes unrelated to gene abundances in stems. High prokaryotic and fungal *nirK* gene abundance in forest canopy soil suggests potential for above-ground denitrification in wet conditions. *nosZ*-I genes found in forest canopy soil and leaves (*E. reunionensis*,* Alsophila glaucifolia*, and *Typha domingensis*) indicate that plants, including forest canopy, may play a significant role in the reduction of N_2_O.

**Supplementary Information:**

The online version contains supplementary material available at 10.1038/s41598-025-12367-y.

## Introduction

In a terrestrial ecosystem, the primary productivity of plants relies on the nitrogen (N) supply. Microorganisms are key players in various N cycling processes. Starting from N fixation^1^ which is regulated by the *nifH* gene. Bacteria and archaea also perform ammonia oxidation (nitrification), including complete ammonia oxidation, and the bacterial and archaeal *amoA* genes regulate the process^2,3^. Denitrification is another crucial step of the microbial N cycle and is regulated by nitrite-reducing (*nirS* and *nirK*) genes and nitrous oxide-reducing (*nosZ*) gene, mainly^4,5^. Fungal denitrification is regulated by fungal *nirK* and *p450nor*^6^. Nitrification and denitrification processes can lead to the production of nitrous oxide (N_2_O), which is a potent greenhouse gas and stratospheric ozone-depleting substance^7–9^.

The organic soils with high nitrate content (NO_3_^−^) are hotspots for N_2_O emissions under drained conditions^10^. Similarly, tropical wetland soils during drought periods have been reported as significant sources of N_2_O^11^ and these emissions are correlated with the functional diversity of microbes and the processes dominated by ammonia-oxidizing archaea^12^. Tropical wetlands and forests are, thus, important components of global N_2_O budgets^13,14^. Even though accurate global estimation of N_2_O fluxes in ecosystems is still a major research challenge, tropical forests are considered a significant source of N_2_O compared to other ecosystems^15–17^ owing to their high organic N content^18^ and high microbial activity in varying soil moisture levels^19^. Field measurements and in situ analyses of various tropical forest ecosystems regarding the N cycle are valuable for developing process-based N cycle models for estimation and mapping of N_2_O emissions^9,17,20^.

The cloud forest ecosystem is one of the least studied tropical ecosystems, especially regarding the microbial N cycle. A cloud forest is a tropical or subtropical forest situated between 600 and 3200 m above sea level (m.a.s.l.), and it is defined by constant or frequent ground-level clouds passing through these forests^21,22^. While just covering 0.4% of the global land surface, these forests show relatively higher species richness and endemism than the rest of the forest ecosystems in the tropics^23^ and high levels of biologically fixed N in their soils^24^. In these forests, nitrogen fixation is associated with the ground cover of mosses and other bryophytes, as they host nitrogen-fixing bacteria^25,26^. The diversity and distribution of microbial communities that fix nitrogen heavily depend on soil moisture^27^. The clouds continuously feed these forests, and the moisture is thus retained; however, the dynamics of nitrogen fixation can be altered during the dry or warm periods. Soil moisture levels, temperature, and soil pH can influence nitrification^28^ and denitrification^29,30^ including N_2_O emissions^31^; however, little is known about the below- and above-ground N cycle processes and N_2_O emissions in tropical cloud forests.

The forest canopy (leaves) has recently been found to be an important area of study regarding N-cycling processes like nitrification^32^ as well as soil N_2_O mitigation^33^. Recent studies on the dynamics of N_2_O fluxes in forest canopy show significant absorption of N_2_O in beech (*Fagus sylvatica*) shoots^34^. Although a study^32^ has shown evidence of canopy nitrification through the presence of *amoA* genes, the above-ground N_2_O consumption process has still not been explained from a microbial perspective. Tree stems are shown to be the weak sources of N_2_O during the dry period in tropical upland forests^35^. During the wet period, tree stems in the tropical lowland rainforest are reported as weak sinks of N_2_O^36^. However, N_2_O reduction mechanisms in forest canopies and tree stems are newly reported phenomena and, thus, are largely unknown. In wetlands, it is shown that plants affect the soil microbiome that regulates N_2_O and N_2_^37^ but the aboveground vegetation role is not yet explained. An integrated analysis of soil and phyllosphere N-cycle microbial communities could possibly shed light on N_2_O transformation in tree canopies^38^ and wetland vegetation.

The canopy soil, as a component of the canopy, has been considered in a few studies regarding the overall N cycling in tropical forest ecosystems^39,40^. Canopy soil is defined as the slowly decomposing material of dead epiphytes, cryptogams (bryophytes, algae, fungi, and lichens), mixed with dust, and plant litter, and harbors a variety of microbial and epiphytic communities that have significant importance in above-ground nutrient cycles^41–44^. In cloud forests, canopy soil is often related to above-ground N cycling, which is regulated differently from ground soil^39^. Globally, the cryptogams are reported as the sources of N_2_O^45^. Studies have shown differences in nutrient levels and microbial diversity in the canopy and ground soils in temperate forests^46^ and tropical rainforests^42,47^. Studies on nutrient cycles in tropical wetlands and peatland cloud forests, particularly integrating soil and vegetation, are scarce. This gap in knowledge hinders our ability to evaluate the sustainability and potential effects of wetland and forest management, climate change, and other disturbances on these ecosystems.

**Fig. 1:**
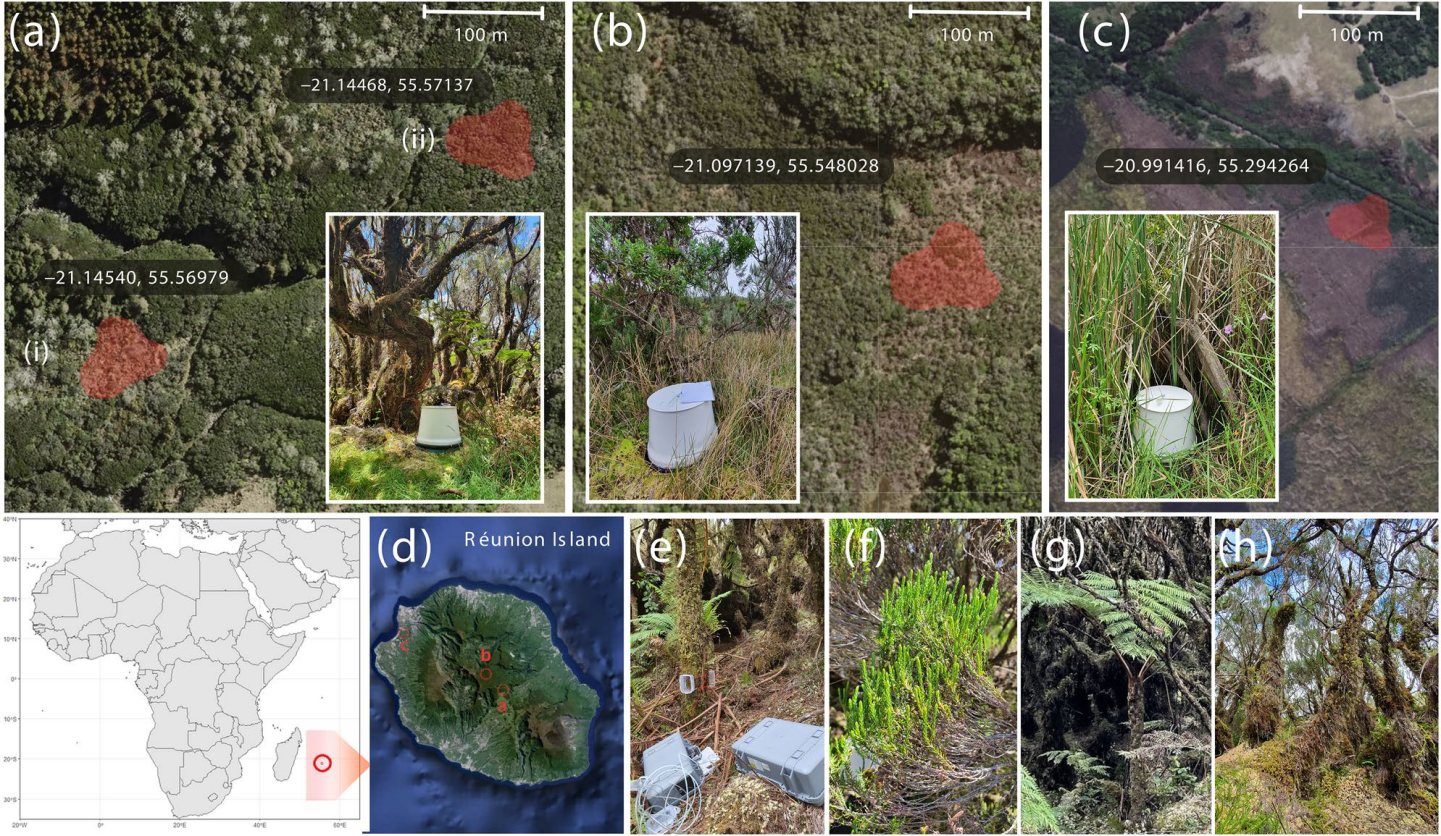
Sampling sites details: **(a)** Plaine des Cafres, - Mixed forest: (i) dominated by endemic fern *Alsophila glaucifolia* (soil, *n* = 6), (ii) dominated by endemic shrub *Erica reunionensis* (soil, *n* = 6). **(b)** Plateau de Thym – Erica forest: featuring *Erica reunionensis* only (soil, *n* = 12). **(c)** Saint Paul wetland with *Typha domingensis* as a dominant plant species (soil, *n* = 6; sediment, *n* = 6). The areas marked in red on the maps indicate where all sampling took place. The embedded pictures of the sites show the soil N_2_O flux sampling using static chambers. **(d)** The locations of study sites on the Réunion Island. **(e)** Tree stem flux measurement with trace gas analyzers. Tree stem fluxes were measured only at cloud forest sites, in addition to the soil flux sampling. **(f)** Leaves of *Erica reunionensis* and **(g)** *Alsophila glaucifolia* were collected from the mixed forest, while only *Erica reunionensis* leaves were collected in the Erica forest (leaves, *n* = 17). **(h)** Canopy soil on the tree stems in the mixed forest comprising decomposing epiphytes (*n* = 19). Stem cores were also obtained from both forests (*n* = 8).

This study aimed to investigate the dynamics of the microbial N cycle and to measure N_2_O fluxes from peat soils and tree stems within tropical cloud forests, as well as from the wetland soils. The study assessed the abundance of functional genes related to the N cycle in two tropical peatland cloud forests (one featuring *Erica reunionensis*, and the other a mix of *E. reunionensis* and *Alsophila glaucifolia*) and a wetland on Réunion Island (Fig. 1). The main novelty of this study is investigating the microbial N cycle in all major forest compartments, including soil, canopy soil, leaves, and tree stem cores. We hypothesized that: (1) Due to different physicochemical conditions, the potential of microbial N cycle processes (nitrification, denitrification, and N fixation) are different in the belowground (peat soil), and above-ground (canopy soil, tree stems, and leaves) compartments of the cloud forests; (2) Archaeal nitrification dominates over denitrification in cloud forest peat soils; (3) The tropical cloud forest peat soils show higher N_2_O emissions and microbial potentials for producing N_2_O than the wetland.

## Results

### Physical and chemical properties of soil

Welch’s ANOVA showed a significant effect of ecosystem type on soil temperature (*F*(2, 15.1) = 423.00, *p* < 0.001). The mean soil temperature at a 10 cm depth was 14.7 °C in mixed forest and 12.9 °C in Erica forest. The wetland mean soil temperature was 21.6 °C, significantly higher than both cloud forest sites (*p* < 0.001). The difference in soil temperature at the depths of 20 cm, 30 cm, and 40 cm followed the same pattern (Table S1). The SWC varied between 0.25 and 0.61 m^3^m^− 3^ in the mixed forest, while it ranged from 0.46 to 0.65 m^3^m^− 3^ in the Erica forest. In the wetland site, it was found to be between 0.87 and 0.90 m^3^m^− 3^. The Games-Howell tests revealed significant differences in SWC between Erica and mixed forests (*p* = 0.021), Erica forest and wetland (*p* < 0.001), and mixed forest and wetland (*p* < 0.001). The mean soil pH was 4.5 and 4.3 in the Erica and mixed forests, respectively. These significantly differed from the wetland samples’ mean pH (7.3, *p* < 0.001).

**Fig. 2:**
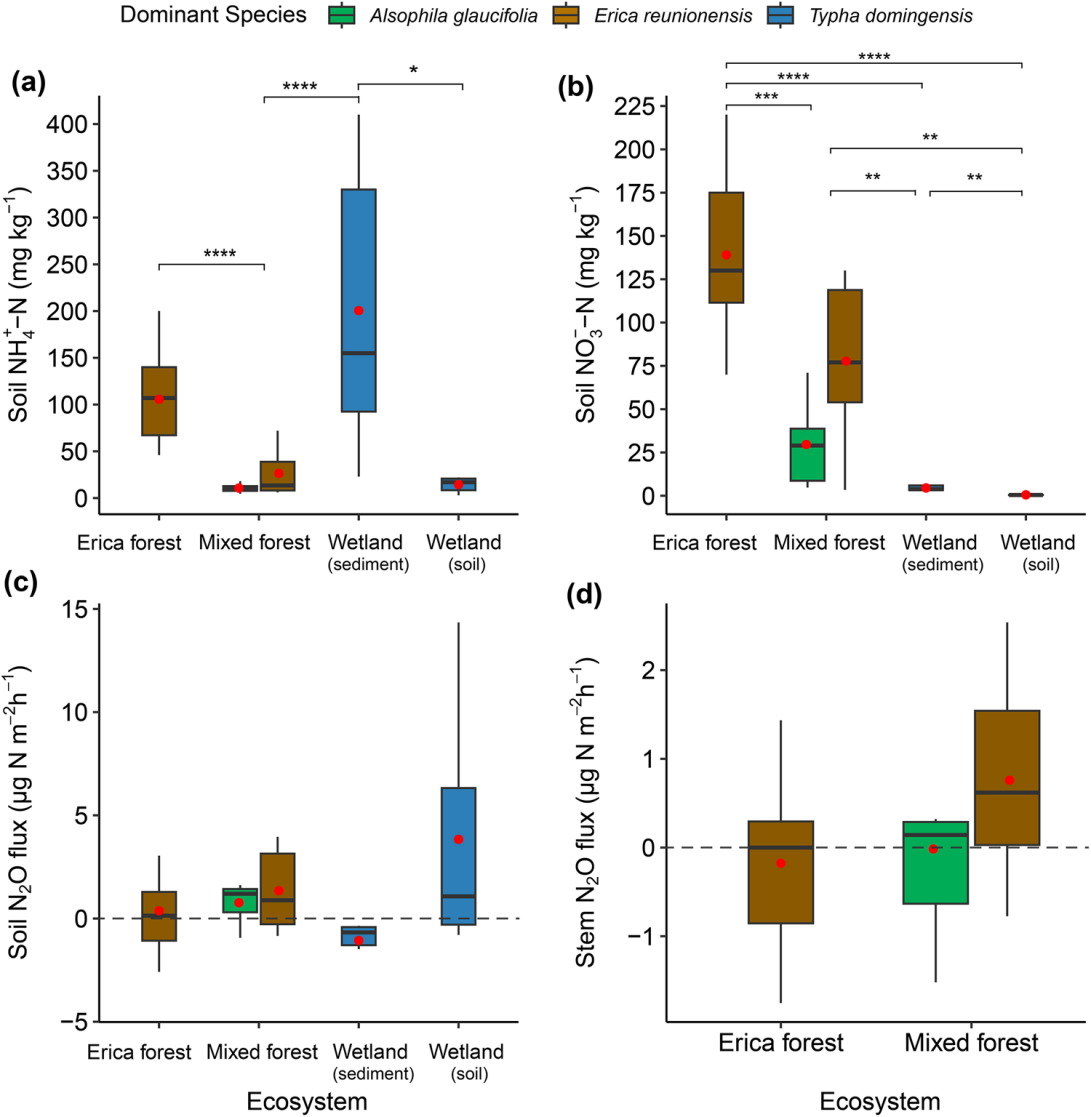
Boxplots of **(a)** soil NH_4_^+^-N levels, **(b)** soil NO_3_^–^-N levels, and **(c)** the soil N_2_O fluxes across the studied ecosystems (*n* = 36). **(d)** Fluxes of N_2_O (µg N m^− 2^ h^− 1^) from the stems of *Erica reunionensis* (*n* = 16, 10 in mixed forest and 6 in Erica forest) and *Alsophila glaucifolia* (*n* = 6) in cloud forests. The colors represent different species dominating the sampling points in a, b, and c. The box represents the interquartile range (IQR) containing the 25th and 75th percentiles of the data distribution. Lines extending from the box (whiskers) represent the range of data within 1.5 times the IQR. The bars inside the box represent the median, and the red dots represent the mean. The significance of the pair-wise relationships is indicated by * (*p* ≤ 0.05), ** (*p* ≤ 0.01), *** (*p* ≤ 0.001), and **** (*p* ≤ 0.0001). Insignificant relationships are not indicated.

In the Erica forest, soil NH_4_^+^-N levels were significantly higher (*p* < 0.001) than in the mixed forest (Fig. 2a, Table S1). Meanwhile, the highest mean value for soil NH_4_^+^-N levels was found in the wetland’s sediment samples, significantly higher than the wetland soil (*p* < 0.05) and the mixed forest (*p* < 0.001). The soil NO_3_^–^-N levels were also significantly higher in Erica forest as compared to mixed forest (*p* < 0.001), and wetland samples (*p* < 0.001). Wetland soil as well as sediments showed the least NO_3_^–^-N levels (Fig. 2b).

### Soil and tree stem fluxes

All sites exhibited low soil N_2_O emissions, with no significant differences between them (Fig. 2c). The mean N_2_O fluxes were 1.06 µg N m^− 2^ h^− 1^ in the mixed forest, 0.37 µg N m^− 2^ h^− 1^ in the Erica forest, and 3.84 µg N m^− 2^ h^− 1^ in the wetland soil. However, the wetland’s water surface was a weak N_2_O sink, and flux was measured at − 1.06 µg N m^− 2^ h^− 1^. We incubated intact soil cores to estimate potential N_2_ and N_2_O fluxes. The respective means of the soil potential N_2_ fluxes were 84.3, 76.4, and 240 µg N m^− 2^ h^− 1^ in the mixed forest, erica forest, and wetland sites (Fig. S1a), and potential N_2_ fluxes were significantly higher in the wetland than in mixed forest soils (*p* < 0.001). The means of the soil potential N_2_O fluxes were 13.1, 6.25, and 10.3 µg N m^− 2^ h^− 1^ in the mixed forest, Erica forest, and wetland sites, respectively, with no significant difference between them (Fig. S1b).

The mean N_2_O fluxes from the stems of *Erica reunionensis* in the Erica forest were − 0.267 µg N m^− 2^ h^− 1^, while in the mixed forest were 0.843 µg N m^− 2^ h^− 1^. The mean N_2_O fluxes from *Alsophila glaucifolia* stems in the mixed forest were − 0.016 µg N m^− 2^ h^− 1^. Overall, the *E. reunionensis* stems showed more variability in the stem N_2_O fluxes from weak sinks to weak sources of N_2_O (Fig. 2d).

### Relationships between soil physicochemical properties

**Fig. 3:**
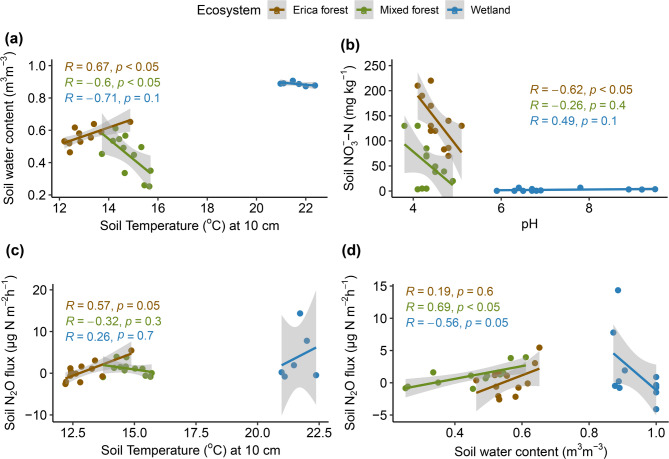
Relationships between **(a)** soil temperature at a depth of 10 cm and SWC (m^3^ m^− 3^) in forest soils, **(b)** pH and the soil NO_3_^–^-N levels in the forest and wetland soils, **(c)** soil temperature at a depth of 10 cm and soil N_2_O flux (µg N m^− 2^ h^− 1^), and **(d)** SWC (m^3^ m^− 3^) and soil N_2_O flux (µg N m^− 2^ h^− 1^) in the forest and wetland soils. Different colors represent the ecosystem type, and shades represent the 95% confidence intervals.

In the two cloud forest sites, the SWC had contrasting relationships with soil temperature: in the Erica-dominated forest, SWC positively correlated with soil temperature at a depth of 10 cm, while in the mixed forest, the correlation was negative (Fig. 3a). Both relationships were statistically significant (*p* < 0.05). In wetland soils, there was no significant correlation. Meanwhile, the low pH significantly correlated with the high NO_3_^−^ values in the Erica forest (*p* < 0.05, Fig. 3b), while there was no significant correlation found between pH and NO_3_^−^ values in wetland samples. Soil temperature had a positive yet statistically insignificant relationship with N_2_O flux in all ecosystems except the Erica forest (*p* = 0.05, Fig. 3c). SWC and soil N_2_O fluxes showed a positive relationship in both forests; however, the relationship was only statistically significant in the mixed forest (*p* < 0.05, Fig. 3d). In the case of the wetland, the relationship between SWC and N_2_O flux was negative (*p* = 0.05).

### Gene abundances and proportions in soil

**Fig. 4:**
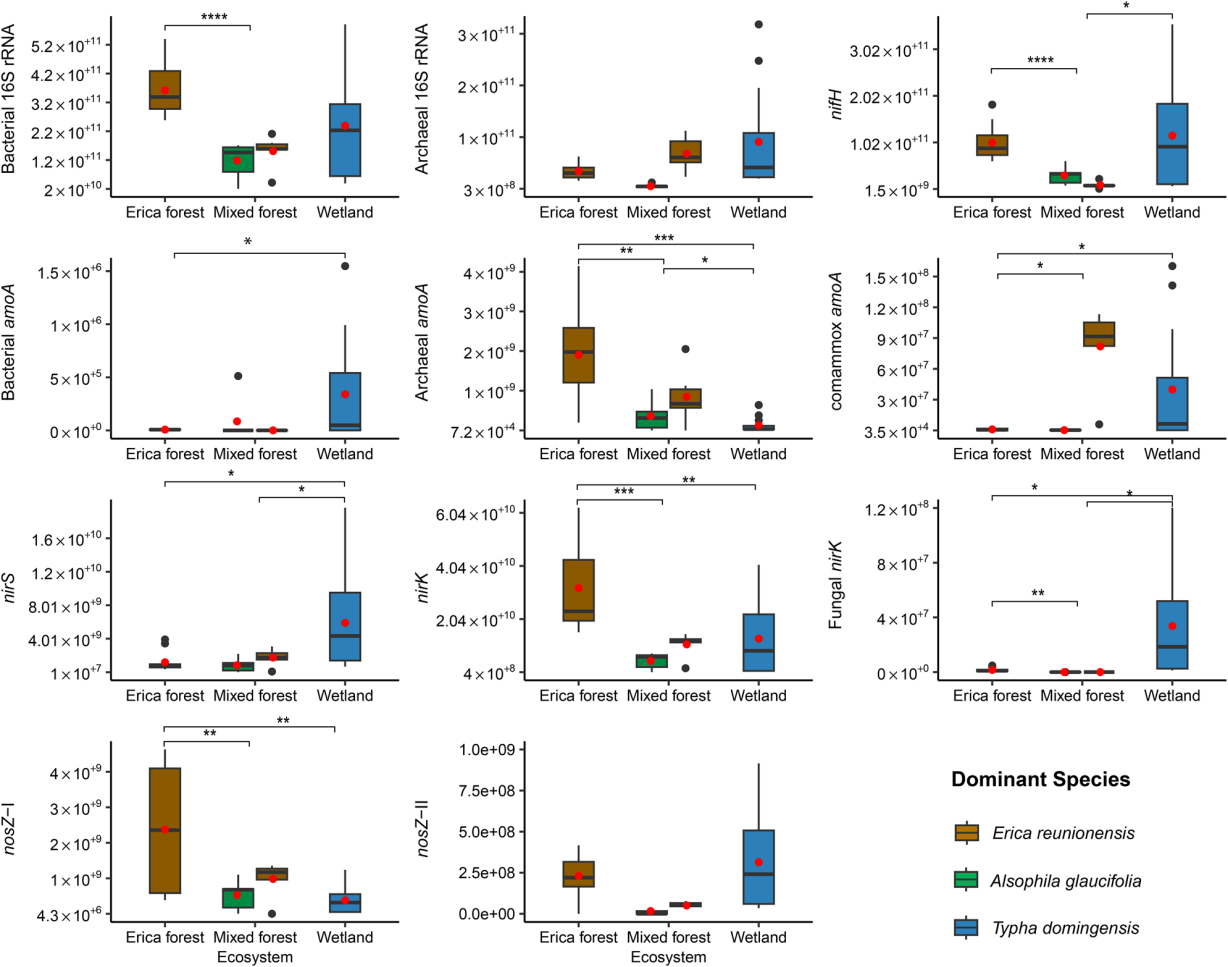
Boxplots of the abundance of different nitrogen-cycling functional genes in the peat soil of the Erica forest (*n* = 12), the Mixed forest (*n* = 12), and the wetland soils/sediments (*n* = 12). The colours of the boxes represent the dominant species found in the site where the soil was obtained. The red dots indicate means, while the bars represent medians. The whiskers show the 95% confidence intervals. The significance of the pairwise relationships is indicated by * (*p* ≤ 0.05), ** (*p* ≤ 0.01), *** (*p* ≤ 0.001), and **** (*p* ≤ 0.0001). Insignificant relationships are not shown.

The post hoc tests conducted on the actual gene abundance values after the Welch’s ANOVA showed that the bacterial 16 S rRNA gene abundance was higher in the Erica forest than in the mixed forest (*p* < 0.001). In the Erica forest, the bacterial 16 S rRNA gene was also higher in proportion (in total prokaryotic abundance) compared to the wetland (*p* < 0.001). The difference in archaeal 16 S rRNA gene abundance was insignificant among all soils (Fig. 4, Table S2). However, the proportion of archaeal 16 S rRNA genes in the total microbial abundance was significantly higher (*p* < 0.001) in wetland samples than in the Erica forest (Table S3).

The erica forest and the wetland soils had a higher abundance (Fig. 4) and proportion of the *nifH* genes than the mixed forest (*p* < 0.001), while the wetland showed the highest *nifH* proportion. The bacterial *amoA* gene abundance was low in forest soils compared to the wetland (*p* < 0.05), with no significant difference among the forest sites. The proportions of the bacterial *amoA* gene were too low for comparison (Table S3). The archaeal *amoA* gene abundance was found to be significantly higher in the Erica forest soil than in the mixed forest (*p* = 0.005) and the wetland (*p* < 0.001). The difference between the mixed forest and the wetland was also statistically significant (*p* = 0.05). The trend was followed by the proportion of archaeal *amoA* gene in total prokaryotic abundance, except that the cloud forest sites had no significant difference among them, but the difference was significant between wetland and cloud forests (*p* < 0.05). The proportion of archaeal *amoA* genes in the total archaeal abundance based on the archaeal 16 S rRNA gene was 5.4% in the Erica forest, 3% in the mixed forest, and 0.3% in the wetland. The mixed forest and the wetland had higher comammox *amoA* gene abundances than the Erica forest soils (*p* < 0.05). The trend was the same for the proportions of comammox *amoA* genes.

The abundance and proportion of the *nirS* gene were significantly higher (*p* < 0.05) in wetland samples compared to cloud forest soils (Fig. 4). The abundance of the *nirK* gene was higher in the Erica forest soil than in the mixed forest and wetland (*p* < 0.001). The proportion of *nirK* genes followed the same trend (Table S3); however, there was no significant difference between the cloud forests, but there was a significant difference between the wetland and the forests (*p* < 0.05). The fungal *nirK* abundance in the wetland samples was significantly higher than in the cloud forest soils (*p* < 0.05).

The *nosZ*-I abundance was significantly higher in the Erica forest soil (*p* < 0.01) than in the mixed forest and wetland (Fig. 4). The *nosZ*-I mean proportions followed the same trend. The soil of mixed forest dominated by *A. glaucifolia* showed the highest abundance and proportion of the *nosZ-*II, significantly different (*p* < 0.05) from the rest of the ecosystems. However, the other ecosystems showed no difference in abundance and proportionality of *nosZ-*II (Table S3).

**Fig. 5:**
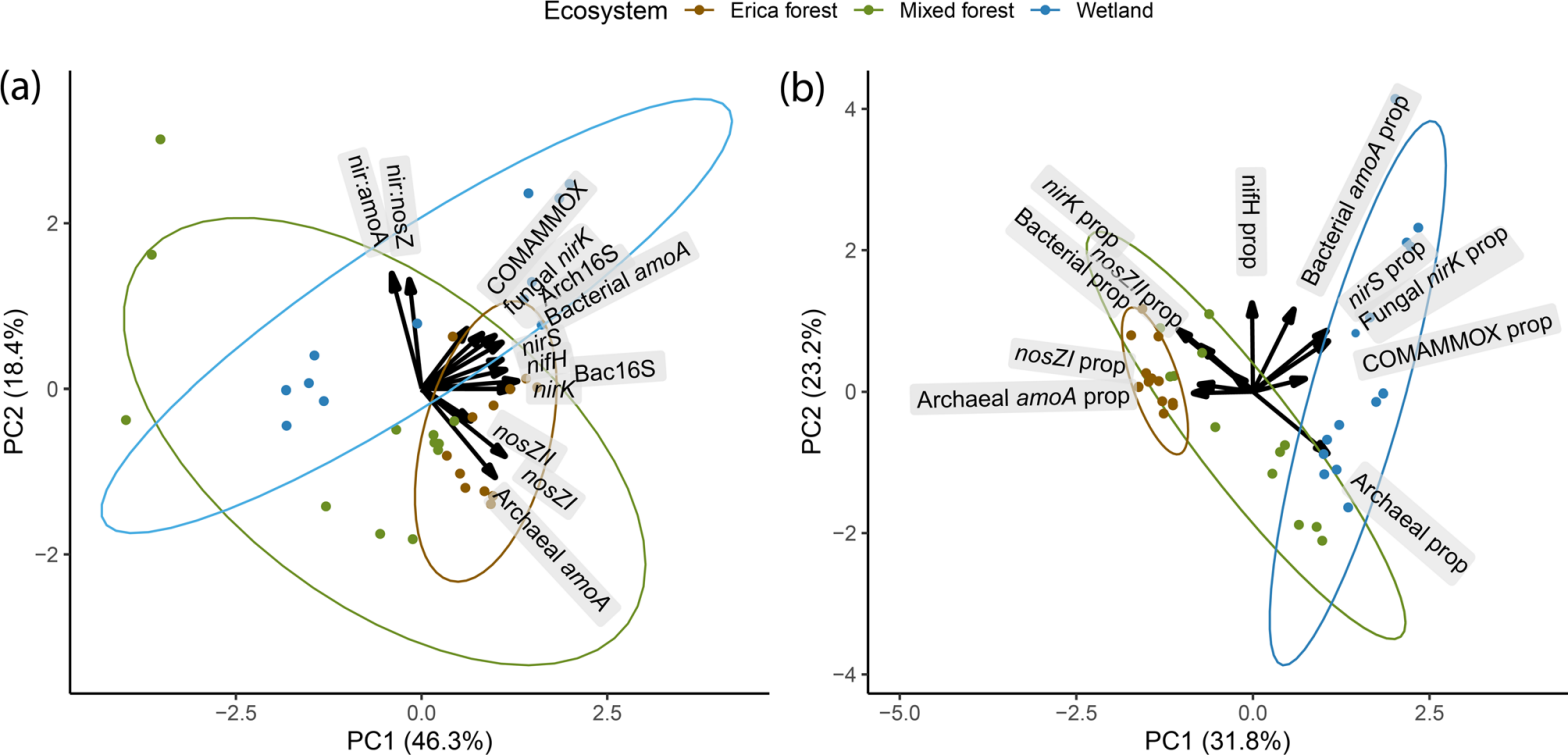
Principal component analysis (PCA) biplots of **(a)** gene abundances and **(b)** the gene proportions in the total prokaryotic abundances in different ecosystems as the groups. The x and y axes represent the first two principal components (PCs), capturing the most significant variation in the data. The length of vectors represents the importance of that gene in explaining the variance captured by the PCs. The ellipses in different colors represent the distribution of samples from different ecosystems (95% confidence intervals).

The overall *nir* gene proportions were highest in the Erica forest soil (8.8%), followed by wetland (6.2%) and mixed forest (5.97%), and the difference was statistically insignificant. The ratio of *nir* to *amoA* genes showed the dominance of *nir* genes over *amoA* genes to be the strongest in the wetland, yet significantly different from the Erica forest only (*p* < 0.05). There was no significant difference in different ecosystems regarding *nir: nosZ*. Principal component analysis (PCA) suggested that the Erica and mixed forest show similarities in their microbial community composition compared to the wetland, where functional genes showed distinct patterns. The ratio of *nir* genes to *amoA* and *nosZ* genes was the highest in the wetland (Fig. 5a). The fungal *nirK* and prokaryotic *nirS* gene proportions also showed the same trend (Fig. 5b).

### Gene abundances and proportions in canopy soil and plant samples

The mean bacterial and archaeal 16 S rRNA gene abundance in canopy soils and other plant samples is given in Table S4. The *nifH*, comammox *amoA*, and *nosZ*-II were not detected in any canopy samples. The bacterial *amoA* genes were only detected in the canopy soil samples. The archaeal *amoA* genes were detected in fern leaves, stems of *E. reunionensis*, and leaves and stalks of *Typha domingensis*. Since the proportions of these genes in total microbial abundance were too low, the difference between the abundances and the proportions in these samples was insignificant.

**Fig. 6:**
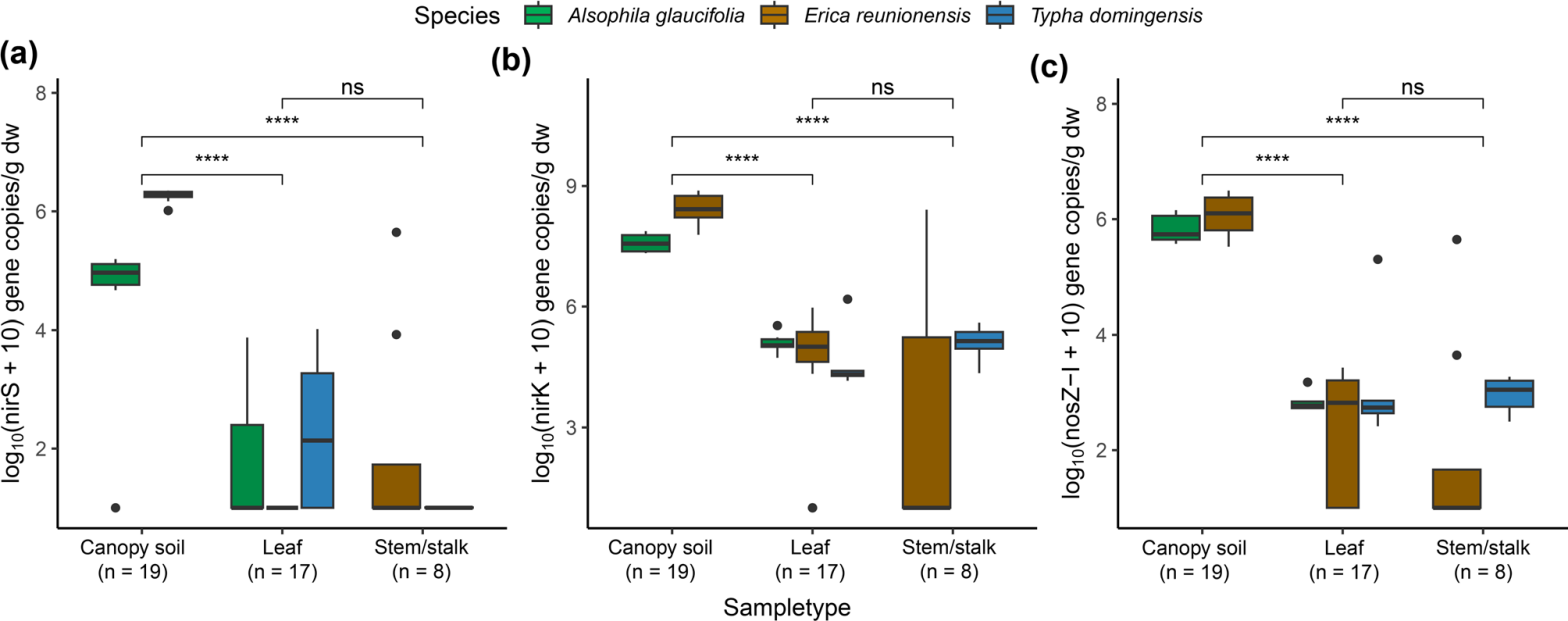
Boxplots of **(a)** *nirS*, **(b)** *nirK*, and **(c)** *nosZ-*I gene abundances in the above-ground samples. The colors represent different plant species. The line within the box shows the median. Dots outside the whiskers represent outliers. The whiskers show the 95% confidence intervals. The significance of the pairwise relationships is indicated by ns (not significant), * (*p* ≤ 0.05), ** (*p* ≤ 0.01), *** (*p* ≤ 0.001), and **** (*p* ≤ 0.0001).

The denitrification genes were detected in most above-ground samples (Fig. 6). The *nirS* gene abundance was found to be significantly greater in the canopy soil as compared to other above-ground samples (*p* < 0.001). The *nirS* genes were not detected in the leaves of *E. reunionensis* and stalks of *T. domingensis*. Meanwhile, the *nirK* genes were abundant in all canopy samples, with canopy soil showing the highest mean value of 2 × 10^8^ copies/g, which were significantly higher than leaves and stem cores (*p* < 0.01). Fungal *nirK* was only detected in the canopy soil. The *nosZ*-I was detected in all the samples taken from the canopy. The highest abundance was found in the canopy soil (*p* < 0.01).

The canopy soil from *E. reunionensis* had a higher abundance of *nirS*, *nirK*, and *nosZ-*I genes than *A. glaucifolia’s* canopy soil (Fig. 6a-c). However, the canopy soil of *A. glaucifolia* showed a higher mean abundance of fungal *nirK* as compared to the *E. reunionensis* canopy soil (*p* < 0.05). There was no significant difference between the *nosZ*-I abundances between leaf samples and typha stalks.

**Fig. 7:**
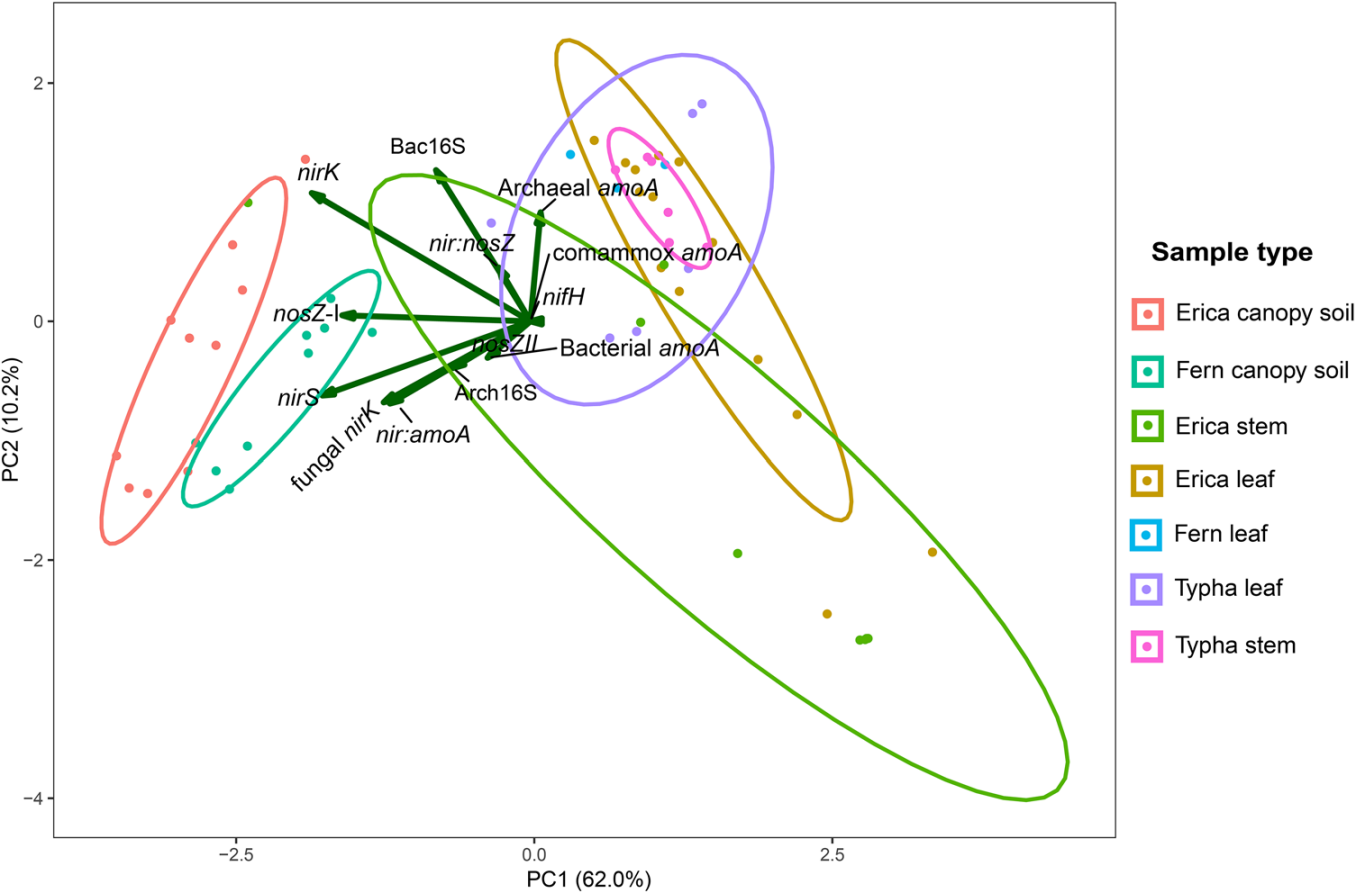
Principal component analyses of gene abundances in the canopy soil and plant leaf samples. The x and y axes represent the first two principal components (PC), capturing the most significant variation in the data. The length of vectors represents the importance of that gene’s abundance in explaining the variance captured by the PCs. The ellipses in different colors represent the sample type distribution (95% confidence intervals).

The PCA analysis demonstrated that the canopy soil samples from *E. reunionensis* and *A. glaucifolia* (fern) formed distinct clusters along both principal components (Fig. 7). Despite this differentiation, the genes responsible for the variation in these components remained consistent, indicating a prevailing presence of denitrification genes (*nirK* and *nosZ*-I) across the various canopy soil samples. However, none of the denitrification gene was found correlating with stem N_2_O fluxes.

### Relationships between gene parameters and soil chemical properties

**Fig. 8:**
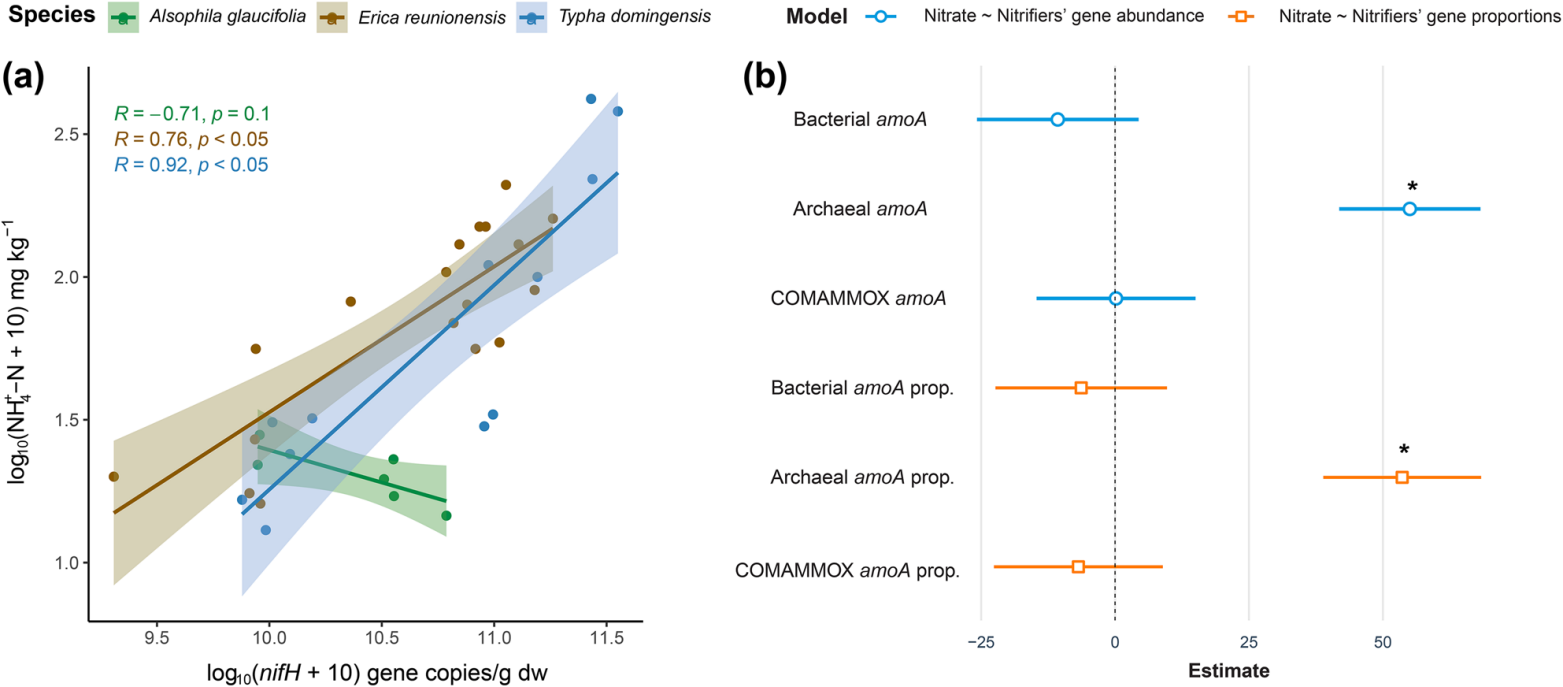
**(a)** Relationship between the *nifH* abundance and the NH_4_^+^-N levels in the soil. **(b)** Multiple regression models between soil NO_3_^–^ and nitrifiers’ abundance and the proportion in the total microbial abundance. Archaeal *amoA* shows the strongest positive relationship with the amount of soil NO_3_^–^-N among all nitrifiers. * represents the *p* < 0.001.

The *nifH* gene abundance positively correlated with soil NH_4_^+^-N levels in the forest soil dominated by *E. reunionensis* (Fig. 8a). The relationship was weakly negative between *nifH* abundance and the ammonium in the forest soil dominated by *A. glaucifolia*. The multiple regression analysis showed a significant relationship between soil NO_3_^–^-N levels and the archaeal *amoA* in terms of both abundance and proportion (Fig. 8b). The estimate values of the regression between archaeal *amoA* and NO_3_^–^-N were significantly higher compared to the regressions with other nitrifying genes (*P* < 0.05). The ground coverage by *Sphagnum* mosses in the cloud forests positively correlated with *nir*:*nosZ* values (Fig. S1). Meanwhile, the gene abundance of the *nosZ*-I positively correlated with the ground percent cover of vascular plants. The mosses’ ground coverage and the soil N_2_O fluxes had a significant negative correlation.

## Discussion

The peat soils in tropical cloud forests, as well as the soil in the wetland ecosystem, were weak sources of N_2_O. Additionally, the surface of the wetland acted as a weak sink for N_2_O (Fig. 2c). Wetland soil showed the least N_2_O: (N_2_ + N_2_O) ratio (Fig. S1c). The tree stems in the tropical cloud forests varied from weak sources to weak sinks of N_2_O (Fig. 2d). There was no significant difference among the soil or tree stem flux values. Our study found high soil NH_4_^+^-N in the peatland cloud forest dominated by *E. reunionensis* (Fig. 2a). The levels of NH_4_^+^-N were significantly correlated with the *nifH* gene abundance in the peat soils dominated by *E. reunionensis* (Fig. 8a). This indicates a high potential for microbial N fixation in the peatland cloud forests. The *nifH* richness in arid and warm ecosystems is usually high^48^ and is related to the high *nifH* gene abundance and nitrogen fixation rates in tropical forest soils^49^. The relatively high *nifH* gene proportions in the Erica forest might suggest a symbiotic relationship between *E. reunionensis* and the *nifH*-containing microbes, as Ericaceae species have been associated with other N-fixing organisms previously^50^. No correlation was found between soil NH_4_^+^-N and *nifH* in soil dominated by *A. glaucifolia* despite possessing high abundance and proportions of *nifH* gene. A possible explanation could be rapid NH_4_^+^ consumption by these ferns; although this has not been previously reported specifically on *A. glaucifolia*, other fern species found in cloud forests have been shown to prefer the consumption of NH_4_^+^ over NO_3_^–^^51^. Furthermore, the symbiotic abilities, transport systems in the root’s plasma membranes, and affinities with the substrates^52^ might also promote the rapid NH_4_^+^ consumption by *A. glaucifolia*.

The high soil NO_3_^–^-N correlated with the archaeal *amoA* abundance and proportions (Fig. 8b). The bacterial *amoA* abundance was not prevalent in the studied ecosystems, and the results indicate that archaeal *amoA* dominated the overall nitrification process. Ammonia-oxidizing archaea (AOA) have been reported to majorly govern the nitrification process in tropical peatlands^12^. Among tropical soils, cloud forest soils have been shown to have the highest abundance of Archaeal *amoA* genes^53^; however, in our sites, this abundance was even higher than what has been reported before. The high soil NO_3_^–^-N also correlated with low soil pH values in both forests (Fig. 3b), which also suggests the active nitrification process, as the increased H^+^ ions generated during the ammonia oxidation result in the acidification of the soil^54,55^. Since AOA prefer and dominate the soils with low pH^28,53,56,57^and the *nirS* and *nirK* expression is reduced in soils with low pH^29,58,59^ we can concur that the efficient archaeal nitrification and a relatively slow *nir*-mediated denitrification process in these peatlands caused the accumulation of soil NO_3_^–^-N. The fungal *nirK* is unaffected by the low pH and can also perform denitrification, but the overall impact would not be substantial, as it constitutes a minor portion of *nirK*-type denitrifiers^60^. Our study found a small abundance of fungal *nirK* in the cloud forest soils, and hence, the role of fungal denitrification in N_2_O emission is minimized. Although the soil pH was low in both cloud forests, the peat soil dominated by *E. reunionensis* showed higher NO_3_^–^-N levels (Fig. 2). This indicates low NO_3_^–^ consumption by *E. reunionensis* due to their restricted ability to utilize NO_3_^[–61^ and their preference for NH_4_^+^ as the major source of inorganic N^62^.

The *nir*:*nosZ* ratio in the soils from both (Erica and mixed) cloud forests was lower than in the soil from the wetland; meanwhile, the *nosZ*-I proportion was significantly higher in the Erica forest soil. The coexistence of *nirS* and *nosZ*-I in the same microbe can favor the complete denitrification^63^ and in tropical soils, N_2_O is often correlated with the *nirK* and fungal *nirK* abundances^64^. We observed very low N_2_O and high N_2_ emissions from the cloud forest peatland soil (Fig. 2c and Fig. S1a). The low pH is reported to inhibit the N_2_O-reducing microbial activity^65^. Therefore, multiple microbial processes can contribute to the high N_2_ flux from cloud forest soils, such as n-DAMO (nitrate-dependent anaerobic methane oxidation), which can yield N_2_ as a byproduct^66^. This can also be supported by the high abundance of NC-10 bacteria (involved in the n-DAMO process) in the peat soils of the same forests^67^. An RNA-based study in a similar ecosystem found the high expression of *nosZ* genes to be the reason behind low soil N_2_O fluxes^68^. In tropical soils, some acidophilic bacteria have been reported to reduce N_2_O at low pH levels^69^. Our N_2_O flux results align with a previous study at a similar elevation range in an Ecuadorian montane forest during the dry season^70^. The soil N_2_O fluxes were also negatively correlated with the ground coverage by mosses in the cloud forests (Fig. S2), which contrasts with the findings of previous studies^71^. The N_2_O fluxes from the soil correlated with the increase in SWC in both cloud forests, and the highest N_2_O flux was observed between the SWC range of 0.6–0.7 m^3^ m^− 3^ (Fig. 3d). This is the same range where many previous studies have observed their peak N_2_O flux in different ecosystems^10,12,72^.

Our study found the highest NH_4_^+^-N levels in the wetland sediments (Fig. 2a), which was positively correlated with the abundance of the *nifH* gene (Fig. 8a). Although *nifH*-containing microbes can perform nitrogen fixation in the sediments^73^ this can not be considered the sole reason behind high NH_4_^+^ in sediment. The prevailing anoxic conditions in the wetland also prevent active nitrification, which results in the accumulation of NH_4_^+^ in its sediments. Another reason can be the extensive use of NH_4_^+^-based fertilizers in the upslope regions of Réunion Island^74^ which can then become part of runoff and finally settle in the lowland wetland sediments. Meanwhile, we found very low NO_3_^–^-N levels in the wetland samples. Although we quantified a substantial abundance of archaeal *amoA* in the wetland, the highest comammox *amoA* found in our study was from the wetland. Typha and phragmites are known for the enormous oxygen release from their roots in the sediments^75,76^. This connective gas flow through the aerenchyma tissues of the *T. domingensis* can aerate the sediment enough to facilitate the comammox.

**Fig. 9:**
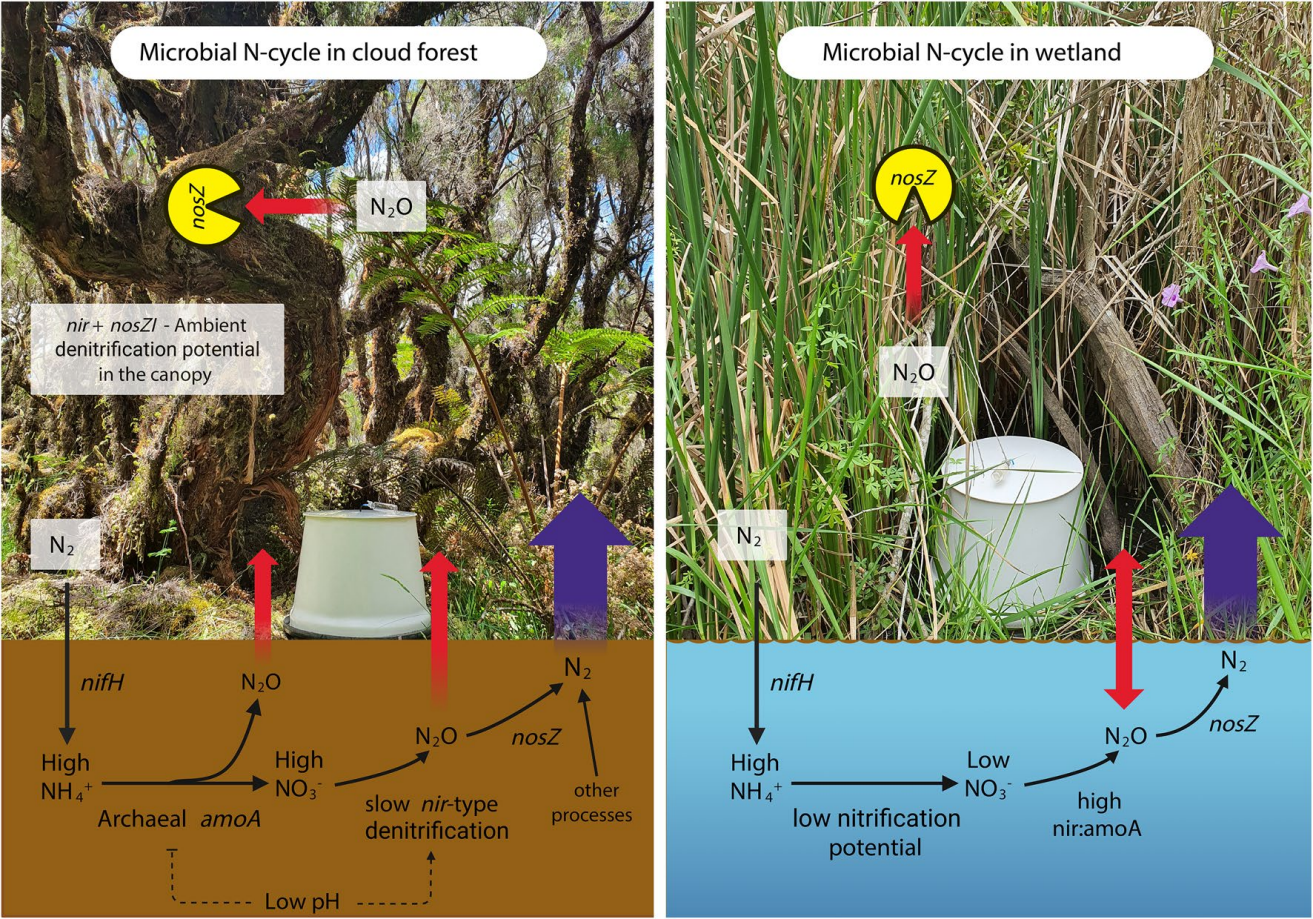
Schematic microbial N cycle in the cloud forest and the wetland site. The arrows represent the potential pathways of the processes in the light of N substrates, N fluxes, and the N cycling microbial genes. The red arrows represent the N_2_O fluxes. In the wetland, the N_2_O flux was positive in soils while it was negative in the wetland surface (hence two-sided arrow). However, the overall amounts were minimal. The large purple arrows represent the N_2_ flux from the soils of the forest and wetland. The difference in the arrows’ size represents the amount of the fluxes.

The low NO_3_^–^-N levels can be attributed to low nitrification as compared to the rapid denitrification since we found a high *nir*:*amoA* ratio in the wetland. Another reason could be the wetland’s fast-growing *T. domingensis*, which can efficiently uptake the NO_3_^–^ from soil and sediment^77^. However, the highest N_2_ fluxes were from the wetland site (Fig. S1a). The dominance of the *nirS* gene and the high *nir* to *amoA* ratio (305 ± 132) suggest that the *nir*-based denitrification actively removed the NO_3_^–^ from the wetland. The *nirS*-type denitrifiers usually dominate the wetland sites^78^ and can also contain the *nosZ* gene^63^. Consequently, in favorable conditions, these denitrifiers can successfully complete denitrification processes. The mean pH level observed in the wetland was recorded at 7.3, which supports the high expression of the *nirS*^59^*norB*, and *nosZ* genes^30^. The dominance of *nir* genes was coupled with a substantial abundance of *nosZ-*I and *nosZ-*II in the wetland. However, the *nir: nosZ* ratio (23.1 ± 3.05) was less than the *nir: amoA* ratio. The high SWC yields prevailing anaerobic conditions, which are conducive to the denitrification process. Typha has also been reported as supporting a high *nosZ* diversity in its rhizosphere^79^. The typha litter also enhances the growth of *nosZ*-containing bacteria in a wetland^80^. We concur that the presence of *Typha* in the wetland might be helpful in the wetland’s N_2_O-reducing efficiency. However, other factors such as n-DAMO can also contribute to the high N_2_ fluxes from the wetland. Our study also revealed a significant presence of *nosZ*-I on both typha leaves and stalks, indicating an additional potential for reducing N_2_O in the wetland vegetation (Fig. 6c). The hypoxic areas of the typha vegetation may contribute to the reduction of absorbed N_2_O from the sediment. The discovery of N_2_O reducers in the vegetative parts of wetlands is unprecedented and requires further research. A schematic N cycle in our studied ecosystems is shown in Fig. 9.

The absence of the *nifH* gene in the canopy samples indicated a lack of microbial N fixation potential in the canopy of cloud forests, contrasting with previous studies in a similar ecosystem, which estimated high N_2_ fixation in the canopy soil during the dry season^81–83^. However, the atmospheric NH_3_ converted to NH_4_^+^ in the presence of cloud droplets or precipitation can be deposited in the canopy^84^. This NH_4_^+^ becomes available for further microbial processes on the plants’ surfaces.

The presence of archaeal *amoA* in canopy samples shows a potential for nitrification in the canopy of cloud forests. The canopy’s microbial nitrification is a reported phenomenon in the forests of the global north, and the process claims 80% of the NO_3_^–^ descending to the soil via throughfall^32,85,86^. Rainfall and cloud droplets might wash the NO_3_^–^ from the canopy to the soil in our studied ecosystems, making the canopy another source of soil NO_3_^–^. The NO_3_^–^ produced through canopy nitrification can also become available for uptake by the epiphytes growing on the canopy soil.

The presence of denitrifiers in the canopy reveals a potential for above-ground denitrification (Fig. 6, Table S4). Canopy soil showed a high abundance of *nirS*, *nirK*, fungal *nirK*, and *nosZ*-I genes. Our study also found a high abundance of *nirK* in stem cores and leaves. The NO_3_^–^ from canopy nitrification^32^ assimilation, and foliar uptake from the atmosphere^87^ can further be utilized and reduced by the denitrifying microbes within the plant’s anoxic or hypoxic parts. This can also occur in the canopy soil when it becomes wet in case of precipitation, and anaerobic conditions on plant surfaces are thus realized. Metatranscriptomics evidence suggests that in such instances where denitrifiers are given anoxic conditions after long periods of oxic conditions, *nirK*-containing microbes exhibit faster rates of NO_3_^–^ reduction. However, during a short-term anoxic situation (e.g., created by a brief spell of rain), only the *nosZ*-II can complete the denitrification^88^. However, our study did not find the *nosZ*-II in the cryptogamic canopy soil. Since our study was conducted in the dry season, where the only source of moisture was the clouds, complete anoxic conditions in the canopy soil were not realized. This can result in the N_2_O emission from the stems. We found stems of *E. reunionensis* as weak sources of N_2_O in the mixed forest where these trees possessed the canopy soil (Fig. 2d). The *E. reunionensis* stems in the Erica forest without any canopy soil were found to be the weak sinks of N_2_O. The absence of *nosZ-*II from the canopy soil can explain this difference. Canopy soil showed a high abundance of *nosZ*-I genes. However, the *nosZ*-I expression will only be highest during long-term anoxic conditions^88^ which was not observed in our study. Such conditions are realized during the rainy season or intense cloud formation in the cloud forests.

The *nosZ*-I was found in the leaf samples of all plant species under study. The N_2_O, which is produced in the soil and transported into different plant compartments^89^ can be reduced by the microbes containing *nosZ*-I found in the hypoxic parts of stems and leaves. The tree stems of the lowland tropical rainforest on the same island were found to be a weak N_2_O sink during the wet season^36^. Our study has confirmed the presence of *nosZ*-I denitrifiers in the cryptogamic canopy soil, providing further evidence that cryptogams can play a significant role in N_2_O uptake^36^. The presence of *nosZ*-I in the stem cores of the cloud forest trees can also explain the N_2_O uptake by *E. reunionensis* stems in our study. The *nosZ*-I microbes in the canopy during the wet season, when anoxic conditions prevail for longer periods on plant surfaces, can reduce the N_2_O emitted by soil and/or canopy soil.

## Materials and methods

### Site description

Two peatland cloud forests were studied on the tropical Réunion Island, France: Plaine des Cafres in Le Tampon municipality (21.145343° S, 55.569692° E) and Plateau de Thym, in Forêt de Bébour region of Saint-Benoît commune (21.097139° S, 55.548028° E) (Fig. 1). Both sites are peatland forests located between 1500 and 1650 m.a.s.l in the montane cloud forest vegetation band^90^.

The Plaine des Cafres’ peatland forest had a mix of endemic shrub species, *E. reunionensis*, and an endemic tree fern species, *Alsophila glaucifolia*. The epiphytic vegetation in this forest was dominated by *Cordyline mauritiana* and various fern species, such as *Hymenophyllum inaequale*, *H*. *capillare*, and *Blechnum attenuatum*. In the understory, *Embelia angustifolia*, *Anthoxanthum odoratum*, and *Cynorkis ridleyi* were the most frequent species. The bryophyte layer was patchy and dominated by *Sphagnum* species. The current study describes this forest as a mixed forest with two sub-sites based on the dominance of either *E. reunionensis* or *A. glaucifolia* (Fig. 1a).

The Plateau de Thym peatland forest had a dominance of *E*. *reunionensis* and *Hubertia ambavilla*. In the understory, the dominating species were *Erica galioides* and *Juncus effusus*, with patchy dominating *Sphagnum* species. The peatland in this forest is approximately 25,000 years old^91^. Peatland forest is described as the erica forest in the current study (Fig. 1b).

The wetland site was situated next to Saint Paul city (20.991416° S, 55.294264° E) at 4 m.a.s.l. The predominant vegetation at the site was *Typha domingensis* (Fig. 1c). Other commonly found species include *Setaria geminata*, aquatic *Lemna* sp, along with exotic *Schinus terebinthifolius* and *Ipomoea cairica* along the edges. In the current study, this site is referred to as a wetland.

All sampling, which included soil, gas fluxes, canopy soil, and plant material, was conducted in November 2022, which marks the early spring dry season on Réunion Island. At every site, 12 measurement points were selected, and soil collars were installed. In the Erica forest, all points were selected in the forest dominated by *E. reunionensis*, while in the mixed forest, 6 points were located in an area dominated by *E. reunionensis* and 6 points in an area dominated by *A. glaucifolia*. At the wetland site, 6 measurement points were located in the soil and 6 points in open water between *Typha* plants. The ground vegetation coverage within the defined area of soil collars was estimated in percentage for all species.

### Soil, canopy soil, and plant sampling

Soil samples (*n* = 36) from all sites were collected from 0 to 10 cm depth for chemical and microbial analysis. Soil collection equipment was disinfected with ethanol between sampling points to prevent cross-contamination, and samples were packed in transparent plastic grip-seal bags. The soil samples for microbial analysis were stored at − 20 °C until DNA extraction. Canopy soil samples (*n* = 19) from the mixed forest were collected from the stem surfaces of *E. reunionensis* and *A. glaucifolia* for microbial analyses. Leaves (*n* = 17) were collected from both cloud forest sites from the different branches of *E. reunionensis* and *A. glaucifolia*. Stem core samples (*n* = 8) were also collected from the stems of *E. reunionensis* at both cloud forest sites. Tree stem samples were taken using a 400 mm length, 5.15 mm diameter, 3-threaded increment borer, and extractor (Haglöf Sweden AB, Langsle, Sweden). From the wetland site, Typha (*Typha domingensis*) leaves (*n* = 6) and stems/stalks (*n* = 6) were collected. The canopy soil and plant samples were placed in filter bags and packed inside sealed plastic bags with active silica gel to eliminate moisture. Silica gel was replaced until all moisture from the samples had been removed successfully. Drying was performed for the long-term preservation of these samples intended for DNA-based analyses^92^.

### Physical and chemical properties of soil

At each measurement point, soil temperature was measured using probes (model CS 107, Campbell Scientific Inc., Logan, UT, USA). The soil water content (SWC) was determined using the ProCheck moisture sensor (Decagon Devices, Inc., USA). Soil chemical analyses were performed at the Estonian Environmental Research Centre in Tartu. The total nitrogen content, total carbon content, and pH level were measured. After extracting with 2 M KCl (1:10 ratio), NH_4_^+^-N and NO_3_^−^-N levels were determined from soil samples using flow-injection analysis according to standard methods^93^.

### N_2_O sampling and flux calculations

To quantify the N_2_O exchange of the soil surface, 65 L polyvinyl chloride chambers (surface area 0.0196 m^2 ^volume 0.065 m^3^and height 0.4 m) were placed to cover the soil surface within pre-installed chamber collars. At the wetland site, the chambers were buoyed in the open water using a foam noodle fixed underneath, and sampling was done without any vegetation inside the chamber. Gas samples were collected from the chambers and injected into 50 ml pre-vacuumed glass bottles during a one-hour measurement period at 20-minute intervals^94^. The N_2_O concentration of all gas samples was tested using two Shimadzu-2014 gas chromatographs equipped with an electron capture detector (GC-ECD), a thermal conductivity detector (GC-TCD), and a Loftfield-type autosampler^95^.

For tree stem N_2_O fluxes, static measurement chambers were installed on the stems of *E. reunionensis* (mixed forest *n* = 10, Erica forest *n* = 6) and *A. glaucifolia* (mixed forest *n* = 6) at one stem height per tree (approximately 20 cm above ground) in both cloud forest sites. The chambers were made from transparent rectangular plastic containers (Lock & Lock, Seoul, South Korea) with bottoms removed to expose the measured area of the tree stem. A neoprene band was glued to the bottom rim of the chamber to make the connection with the tree stem surfaces airtight. Each chamber was closed with an airtight removable lid connected to the gas analyzers. The gas concentrations were measured by circulating air in a closed loop between the chamber and a portable N_2_O/H_2_O trace gas analyzer (LI-7820, Li-Cor Biosciences, Lincoln, NE, USA) over 10 min. For soil and stem flux calculations, we determined the slope of the least-squares linear regression of the change in N_2_O concentrations in the chamber headspace over the measurement time. Detailed equations used for the calculations can be found in the study by Ranniku et al. 2024^96^. The quality of each measurement session was validated using the adjusted R^2^ value of the linear regression for the CO_2_ measurements, also determined using the gas chromatograph, which certifies chamber closure quality. Flux values were accepted if the R^2^ value of the CO_2_ slope exceeded 0.9.

In addition, intact soil cores (diameter 6.8 cm) from the topsoil layer (0–10 cm) were obtained after the gas sampling was complete for each site. Potential N_2_ and N_2_O fluxes were measured from these soil cores in the laboratory using the helium atmosphere method for soil incubation^97,98^.

### DNA extraction

Before DNA extraction, the canopy soil and the plant samples were crushed using a coffee grinder. The grinder parts were sterilized with 70% ethanol between every sample. The DNeasy PowerSoil Pro kit (Qiagen, Hilden, Germany) was used to extract DNA from 0.25 g of the soil and 0.12 g of canopy soil and plant material, following the instructions provided by the manufacturer. All samples (soil, canopy soil, stem, and leaves) were homogenized with lysis buffer using Precellys 24 Homogenizer (Berlin Technologies, Montigny-le-Bretonneux, France) at 5000 rpm for 20s. In the case of canopy soil and plant samples, the amount of lysis buffer (CD1) was increased up to 50–70% to maximize the amount of lysate. The concentration and quality of the extracted DNA were measured using a Tecan AG Infinite M200 spectrophotometer before storage at − 20 ºC.

### Quantitative polymerase chain reaction (qPCR)

To determine the bacterial and archaeal abundance in all samples, the qPCR assay of 16 S ribosomal RNA (rRNA) genes was performed using RotorGene^®^ Q equipment (Qiagen, Valencia, CA, USA). The abundance of N-fixing microbes was determined by quantifying the *nifH* gene, which encodes the nitrogenase enzyme, using qPCR. Similarly, nitrifier abundance was determined by quantifying bacterial and archaeal *amoA* and the comammox (complete ammonia oxidizers) *amoA* (encoding ammonia monooxygenase) gene. The abundance of nitrite-reducing denitrifiers was determined by quantifying the *nirS* (gene encoding cytochrome cd1 nitrite reductase) and the *nirK* (gene encoding the copper-containing nitrite reductase). The nitrous oxide reductase-encoding gene, *nosZ* (clade I and II), was quantified to determine the abundance of microbes involved in the final denitrification step. The primers and the qPCR program profiles are shown in Table S5. Negative controls were included in every qPCR run.

The qPCR data was analyzed using RotorGene Series Software (version 2.0.2, Qiagen, Hilden, Germany) and LinRegPCR program (version 2020.0). Gene copy numbers were determined from the samples’ threshold cycles and corrected by the dry weight%, expressed as gene copies per gram of dry weight of the sample (copies g^-1^ dw).

### Statistical data analyses

Jamovi (version 2.4.8)^99^ was used for the basic descriptive analyses and was used to perform the normality tests on the data by employing the Shapiro-Wilks tests and drawing the histograms and quantile-quantile (Q-Q) plots. The variables were analyzed based on different ecosystem types and the dominant tree species in the ecosystem as factors. In RStudio (R version 2024.4.1.748)^100^a *ggstatsplot* package^101^ was used to perform one-way ANOVA (Welch) to compare the means of different variables in different ecosystems. Games-Howell tests as *post hoc* pairwise comparisons were employed to account for the violation of variance homogeneity. Principal component analysis (PCA) plots were created using the *ggbiplot* package in RStudio, while the rest of the plots were created using the *ggplot2* package. The gene abundances and soil NH_4_^+^ values were log_10_ transformed to obtain normality before being used in different statistical analyses.

## Electronic supplementary material

Below is the link to the electronic supplementary material.

Supplementary Material 1

## Data Availability

The datasets generated during and/or analyzed during the current study are available from the corresponding author upon reasonable request.
