# Supplementary material for: Nitrogen cycling genes abundance in soil and aboveground compartments of tropical peatland cloud forests and a wetland on Réunion Island
Source: Sci Rep. 2025 Jul 25;15:27155. doi: 10.1038/s41598-025-12367-y (PMC12297492; doi:10.1038/s41598-025-12367-y)
Supplement: Supplementary file 1 — Supplementary Material 1 [file 41598_2025_12367_MOESM1_ESM.docx]

**Supplementary material**

**Nitrogen cycling genes abundance in soil and aboveground compartments of tropical peatland cloud forests and a wetland on Réunion Island**

**Fahad Ali Kazmi**^1*^, Ülo Mander^1^, Reti Ranniku^1,2^, Maarja Öpik^3^, Kersti Püssa^3^, Kaido Soosaar^1^, Kuno Kasak^1,4^, Mohit Masta^1^, Claudine Ah-Peng^5,6^, Mikk Espenberg^1^

^1^ Department of Geography, University of Tartu, Tartu, 51003 Estonia

^2^ Department of Biological and Agricultural Engineering, University of Arkansas, Fayetteville, AR 72701, USA

^3^ Department of Botany, University of Tartu, Tartu, 50409 Estonia

^4^ Department of Environmental Science, Policy and Management, University of California, Berkeley, USA

^5^ UMR PVBMT, Université de La Réunion, 97410 Saint-Pierre, La Réunion, France

^6^ OSU-Réunion, Université de La Réunion, 97400 Saint-Denis, La Réunion, France

**Corresponding author:** Fahad Ali Kazmi (fahad.ali.kazmi@ut.ee)

ORCID : <https://orcid.org/0000-0001-8088-961X>

# **Supplementary Material**

**
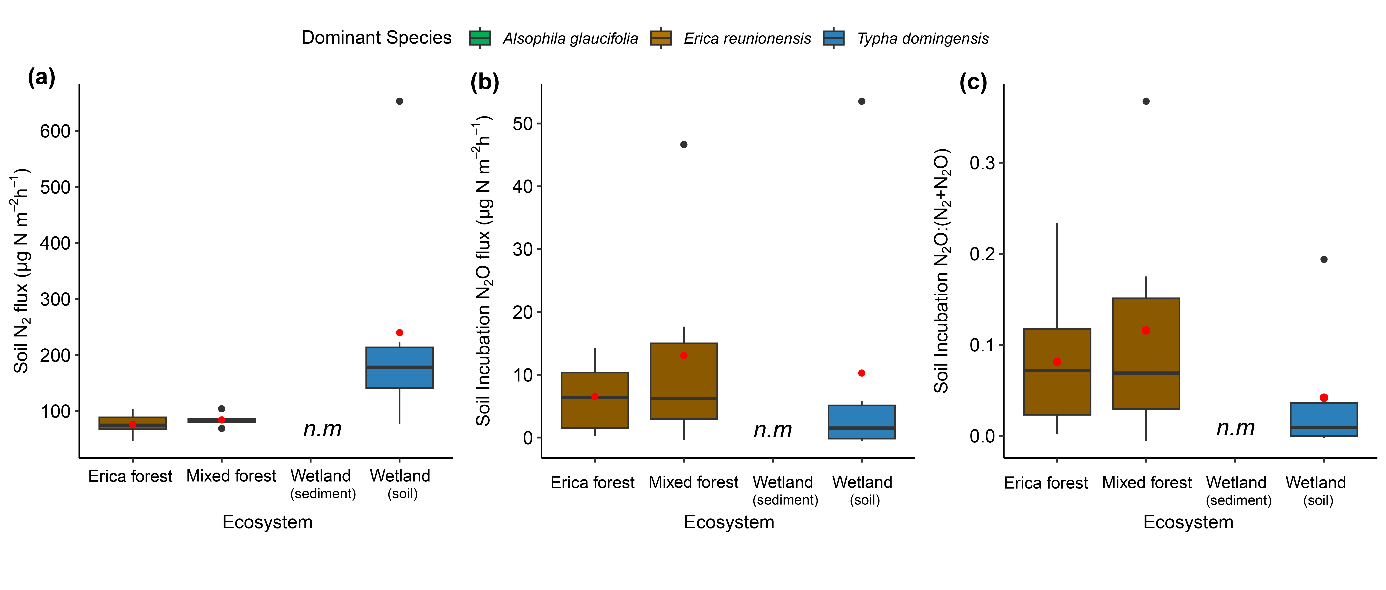
**

**Supplementary Fig. 1**. Boxplots of incubation **(a)** N_2_ flux, **(b)** N_2_O flux, and **(c)** N_2_O: N_2_+N_2_O ratio (n=18). The colors represent different species dominating the sampling points. The box represents the interquartile range (IQR) containing the 25^th^ and 75^th^ percentiles of the data distribution. Lines extending from the box (whiskers) represent the range of data within 1.5 times the IQR. The bars represent the median, the red dots represent the mean, and the black dots represent the outliers. Wetland sediments were not incubated, hence marked as (n.m – not measured).

**
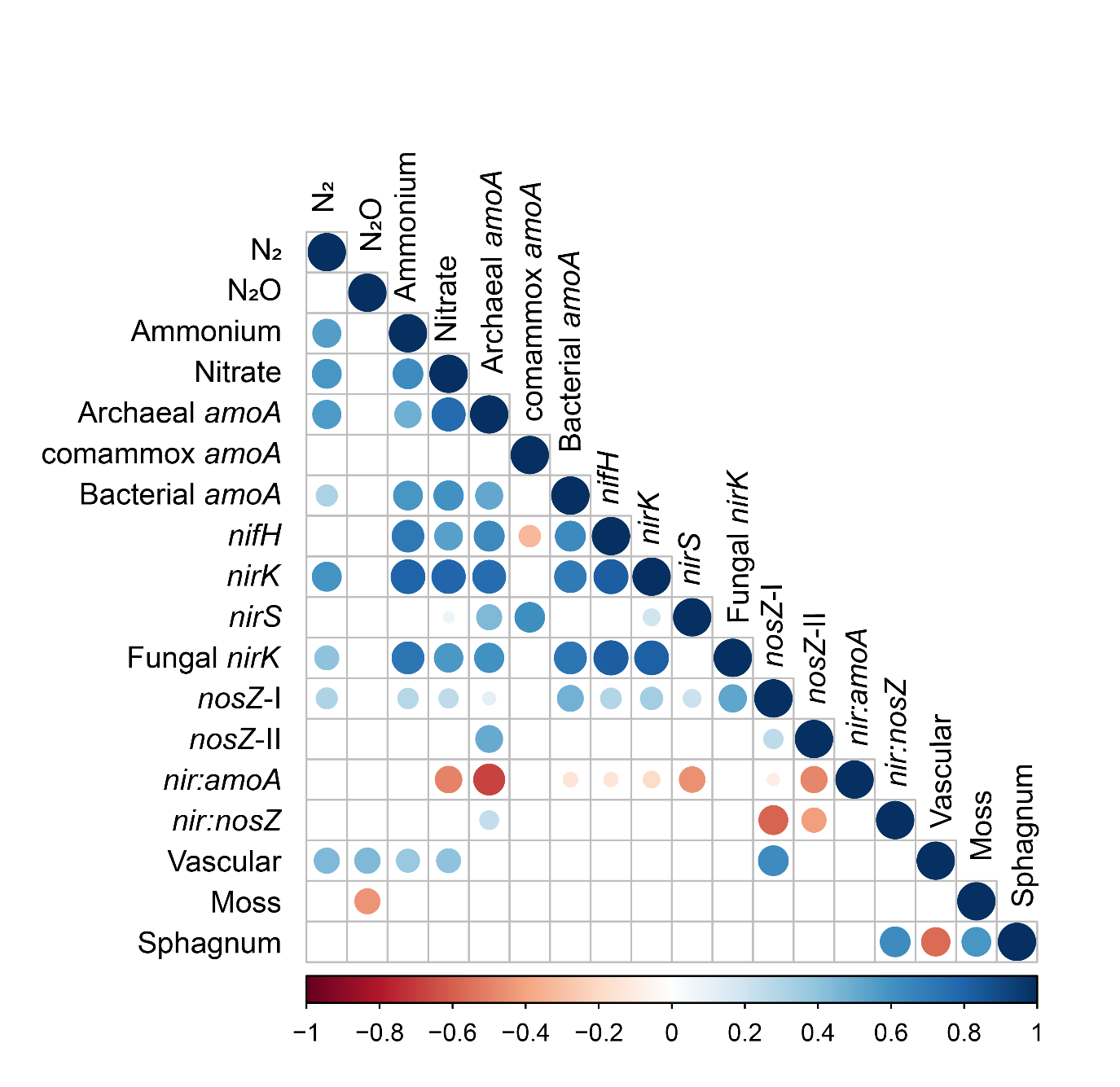
Supplementary Fig. 2.** The Spearman correlation matrix of soil N fluxes, N substrates, the abundances of different functional genes and their ratios in soils (n = 36), and ground vegetation coverage. The blank boxes indicate insignificance in the correlation.

| **Ecosystem** | **pH** | **Soil T10**  (°C) | **Soil T20** (°C) | **Soil T30** (°C) | **Soil T40** (°C) | **SWC (**m^3^m^−3^) | **C**:**N** | NH_4_^+^-N (**mg/kg)** | NO_3_^–^-N (**mg/kg)** |
| --- | --- | --- | --- | --- | --- | --- | --- | --- | --- |
| Erica forest | 4.55 ± 0.08 | 12.9 ± 0.22 | 11.7 ± 0.17 | 11.8 ± 0.18 | 11.7 ± 0.16 | 0.561 ± 0.01 | 12.8 ± 0.59 | 106 ± 13.7 | 139 ± 14.2 |
| Mixed forest | 4.33 ± 0.08 | 14.8 ± 0.18 | 12.9 ± 0.10 | 13.2 ± 0.08 | 13.1 ± 0.09 | 0.446 ± 0.03 | 23.5 ± 3.65 | 18.6 ± 5.80 | 53.7 ± 12.9 |
| Wetland | 7.30 ± 0.35 | 21.6 ± 0.21 | 21.1 ± 0.16 | 20.5 ± 0.69 | 21.2 ± 0.17 | 0.943 ± 0.01 | 15.1 ± 0.63 | 107 ± 41.8 | 2.53 ± 0.68 |

**Supplementary Table 1. The mean values of the physical and chemical parameters of the soil based on the ecosystem.** **Mean values ± standard error of means.**

# **Supplementary Table 2. Abundances of different functional genes in soil samples (gene copies/g of dry weight). Mean values ± standard error of means.**

| Ecosystem | **Erica forest** |  | **Mixed forest** | |  | **Wetland** |
| --- | --- | --- | --- | --- | --- | --- |
| Dominant Species | *Erica reunionensis* |  | *Alsophila glaucifolia* | *Erica reunionensis* |  | *Typha domingensis* |
| **Archaeal 16S rRNA** | 3.42×10^10^ ± 4.27×10^9^ |  | 5.25×10^9^ ± 1.64×10^9^ | 6.81×10^10^ ± 1.37×10^10^ |  | 9.10×10^10^ ± 2.98×10^10^ |
| **Bacterial 16S rRNA** | 3.62×10^11^ ± 2.56×10^10^ |  | 1.19×10^11^ ± 2.58×10^10^ | 1.51×10^11^ ± 2.35×10^10^ |  | 2.39×10^11^ ± 5.82×10^10^ |
| **Archaeal *amoA*** | 1.91×10^9^ ± 3.10×10^8^ |  | 3.63×10^8^ ± 1.58×10^8^ | 8.49×10^8^ ± 2.83×10^8^ |  | 1.30×10^8^ ± 5.69×10^7^ |
| **Comammox *amoA*** | 1.06×10^6^ ± 3.3×10^5^ |  | 2.0×10^5^ ± 7.5×10^4^ | 8.19×10^7^ ± 1.6×10^7^ |  | 3.99×10^7^ ± 1.71×10^7^ |
| **Bacterial *amoA*** | 7.3×10^3^ ± 1.2×10^3^ |  | 8.5×10^4^ ± 8.5×10^4^ | 4.6×10^2^ ± 1.3×10^2^ |  | 3.4×10^5^ ± 1.5×10^5^ |
| ***nifH*** | 1.01×10^11^ ± 1.07×10^10^ |  | 3.05×10^10^ ± 8.0×10^9^ | 9.94×10^9^ ± 2.8×10^9^ |  | 1.16×10^11^ ± 3.52×10^10^ |
| ***nirK*** | 3.20×10^10^ ± 4.77×10^9^ |  | 4.69×10^9^ ± 1.26×10^9^ | 1.09×10^10^ ± 1.86×10^9^ |  | 1.30×10^10^ ± 4.51×10^9^ |
| ***nirS*** | 1.19×10^9^ ± 3.43×10^8^ |  | 8.40×10^8^ ± 3.31×10^8^ | 1.76×10^9^ ± 4.15×10^8^ |  | 5.90×10^9^ ± 1.66×10^9^ |
| **Fungal *nirK*** | 1.61×10^6^ ± 4.0×10^5^ |  | 5.7×10^3^ ± 2.7×10^3^ | 3.7×10^3^ ± 9.9×10^2^ |  | 3.37×10^7^ ± 1.11×10^7^ |
| ***nosZ*-I** | 2.38×10^9^ ± 5.09×10^8^ |  | 5.34×10^8^ ± 1.78×10^8^ | 9.87×10^8^ ± 2.07×10^8^ |  | 3.83×10^8^ ± 1.07×10^8^ |
| ***nosZ*-II** | 2.30×10^8^ ± 3.60×10^7^ |  | 1.81×10^9^ ± 1.46×10^9^ | 5.04×10^7^ ± 1.12×10^7^ |  | 3.14×10^8^ ± 8.29×10^7^ |
| ***nir:amoA*** | 25.8 ± 6.89 |  | 99.1 ± 80.5 | 68 ± 51.7 |  | 305 ± 132 |
| ***nir:nosZ*** | 28.9 ± 8.58 |  | 16.2 ± 10.6 | 83.6 ± 71.6 |  | 23.1 ± 3.05 |

**Supplementary Table 3. Proportions of the functional genes in the total microbial abundance (bacteria & archaea) in the soil samples (%). Mean percentage values ± standard error of means.**

| Ecosystem | **Erica forest** | **Mixed forest** | | **Wetland** |
| --- | --- | --- | --- | --- |
| Dominant Species | *Erica reunionensis* | *Alsophila glaucifolia* | *Erica reunionensis* | *Typha domingensis* |
| **Archaeal proportion** | 8.64 ± 0.98 | 3.93 ± 0.73 | 31.3 ± 3.13 | 26.1 ± 2.33 |
| **Bacterial proportion** | 91.4 ± 0.98 | 96.1 ± 0.73 | 68.7 ± 3.13 | 73.9 ± 2.33 |
| **Archaeal *amoA*** | 0.45 ± 0.05 | 0.218 ± 0.08 | 0.33 ± 0.1 | 0.05 ± 0.01 |
| **Comammox *amoA*** | 2.2 × 10^-4^ ± 6.6 × 10^-6^ | 2.47 × 10^-4^ ± 5.03 × 10^-5^ | 0.03 ± 0.005 | 0.01 ± 0.005 |
| **Bacterial *amoA*** | 1.95 × 10^-6^ ± 3.42 × 10^-7^ | 4.85 × 10^-5^ ± 4.85 × 10^-5^ | 2.07 × 10^-7^ ± 5.53 × 10^-8^ | 9.45 × 10^-5^ ± 4.90 × 10^-5^ |
| ***nifH*** | 25.7 ± 2.17 | 26 ± 4.19 | 4.33 ± 0.84 | 27.7 ± 4.06 |
| ***nirK*** | 7.8 ± 0.79 | 3.35 ± 0.51 | 4.77 ± 0.48 | 2.9 ± 0.64 |
| ***nirS*** | 0.286 ± 0.06 | 0.51 ± 0.17 | 0.73 ± 0.16 | 1.82 ± 0.29 |
| ***nosZ*-I** | 0.71 ± 0.17 | 0.33 ± 0.10 | 0.4 ± 0.09 | 0.11 ± 0.01 |
| ***nosZ*-II** | 0.0604 ± 0.009 | 1.07 ± 0.80 | 0.02 ± 0.004 | 0.09 ± 0.009 |

# **Supplementary Table 4. Abundances of different functional genes in canopy samples (gene copies/g of dry weight). Mean values ± standard error of means. nd – under detection limit**

|  | **Erica forest** | | **Mixed forest** | | | | **Wetland** | |
| --- | --- | --- | --- | --- | --- | --- | --- | --- |
|  | Erica leaf | Erica stem | Canopy soil | Erica leaf | Fern leaf | Erica stem | Typha leaf | Typha stalk |
| **Archaeal 16S rRNA** | 2.85×10^6^ ± 1.20×10^6^ | 6.75×10^7^ ± 1.99×10^7^ | 3.56×10^7^ ± 1.17×10^7^ | 2.22×10^5^ ± 3.43×10^5^ | 6.46×10^6^ ± 1.09×10^7^ | 2.57×10^8^ ± 2.35×10^8^ | 7.51×10^5^ ± 4.2×10^5^ | 2.40×10^4^ ± 6.12×10^3^ |
| **Bacterial 16S rRNA** | 2.19×10^10^ ± 7.42×10^9^ | 4.35×10^8^ ± 1.31×10^8^ | 4.30×10^9^ ± 9.8×10^8^ | 3.61×10^9^ ± 1.80×10^9^ | 2.26×10^8^ ± 1.32×10^8^ | 2.46×10^9^ ± 2.46×10^9^ | 3.02×10^9^ ± 1.27×10^9^ | 1.13×10^9^ ± 2.36×10^8^ |
| **Archaeal *amoA*** | nd | nd | 9.11×10^2^ ± 6.11×10^2^ | 7.06×10^3^ ± 7.06×10^3^ | 1.05×10^5^ ± 4.86×10^3^ | 807 ± 807 | 5.02×10^4^ ± 3.17×10^4^ | 602 ± 264 |
| **Comammox *amoA*** | nd | nd | nd | nd | nd | nd | nd | nd |
| **Bacterial *amoA*** | nd | nd | 8.95×10^4^ ± 8.84×10^4^ | nd | nd | nd | nd | nd |
| ***nifH*** | nd | nd | 0 | nd | nd | nd | nd | nd |
| ***nirK*** | 3.65×10^5^± 1.54×10^5^ | 4.84×10^5^± 4.45×10^5^ | 2.09×10^8^ ± 8.30×10^7^ | 8.49×10^4^ ± 3.80×10^4^ | 1.41×10^5^ ± 7.71×10^4^ | 5.13×10^7^ ± 5.13×10^7^ | 2.70×10^5^ ± 2.49×10^5^ | 1.77×10^5^ ± 5.71×10^4^ |
| ***nirS*** | nd | 2.80×10^3^ ± 2.80×10^3^ | 1.02×10^6^ ± 1.15×10^5^ | nd | 1.17×10^3^ ± 2.30×10^3^ | 8.84×10^4^ ± 8.84×10^4^ | 2.35×10^3^ ± 1.64×10^3^ | 0.0886 ± 0.05 |
| **Fungal *nirK*** | nd | nd | 8.12×10^4^ ± 1.40×10^4^ | nd | nd | nd | nd | nd |
| ***nosZ*-I** | 9.30×10^2^ ± 3.34×10^2^ | 1.47×10^3^ ± 1.47×10^3^ | 1.20×10^6^ ± 3.42×10^5^ | 1.12×10^3^ ± 3.46×10^2^ | 6.31×10^2^ ± 3.00×10^2^ | 8.87×10^4^ ± 8.87×10^4^ | 3.39×10^4^ ± 3.34×10^4^ | 1.09×10^3^ ± 2.63×10^2^ |
| ***nosZ*-II** | nd | nd | nd | nd | nd | nd | nd | nd |

**Supplementary Table 5. Primers and qPCR program details for the functional genes studied.**

| **Target gene** | **Primer** | **Primer reference** | **Amplicon size (bp)** | **Primer concentration (µM)** | **qPCR program** |
| --- | --- | --- | --- | --- | --- |
| Bacterial 16S rRNA | Bact517F | [1] | 530 | 0,6 | 95°C 10 min; 35 cycles: 95°C 30 s; 60°C 45 s; 72°C 45 s |
|  | Bact1028R | [2] |  |  |  |
| Archaeal 16S rRNA | Arc519F | [3] | 393 | 0,6 | 95°C 10 min; 45 cycles: 95°C 15 s; 56°C 30 s; 72°C 30 s |
|  | Arch910R |  |  |  |  |
| *nirS* | nirSCd3af | [4] | 431 | 0,8 | 95°C 10 min; 45 cycles: 95°C 15 s; 55°C 30 s; 72°C 30s, 80°C 30 s^a^ |
|  | nirSR3cd |  |  |  |  |
| *nirK* | nirK876 | [5] | 165 | 0,8 | 95°C 10 min; 45 cycles: 95°C 15 s; 58°C 30 s; 72°C 30s, 80°C 30 s^a^ |
|  | nirK1040 |  |  |  |  |
| *nosZI* | nosZ2F | [6] | 267 | 0,6 | 95°C 10 min; 45 cycles: 95°C 15 s, 60°C 30 s, 72°C 30 s, 80°C 30 s^a^ |
|  | nosZ2R |  |  |  |  |
| *nosZII* | nosZIIF | [7] | ~700 | 0,6 | 95°C 10 min; 45 cycles: 95°C 30 s, 54°C 45 s, 72°C 45 s, 80°C 45 s^a^ |
|  | nosZIIR |  |  |  |  |
| Bacterial *amoA* | amoA-1F | [8] | 491 | 0,8 | 95°C 10 min; 45 cycles: 95°C 30 s; 57°C 45 s; 72°C 45 s |
|  | amoA-2R |  |  |  |  |
| Archaeal *amoA* | CrenamoA 23F | [9] | ~600 | 0,8 | 95°C 10 min; 45 cycles: 95°C 30 s; 55°C 45 s; 72°C 45 s |
|  | CrenamoA 616R |  |  |  |  |
| COMAMMOX *amoA* | comamoA AF | [10] | 436 | 0,8 | 95°C 10 min; 40 cycles: 95°C 15 s, 55°C 30 s, 72°C 30 s |
|  | comamoA SR |  |  |  |  |
| *nifH* | Ueda19F | [11] | 390 | 0,8 | 95°C 10 min; 45 cycles: 95°C 30 s, 53°C 45 s, 72°C 45 s |
|  | Ueda407R |  |  |  |  |

# **References**

1. Liu, Z., Lozupone, C., Hamady, M., Bushman, F. D. & Knight, R. Short pyrosequencing reads suffice for accurate microbial community analysis. *Nucleic Acids Res.* **35**, e120 (2007).

2. Dethlefsen, L., Huse, S., Sogin, M. L. & Relman, D. A. The Pervasive Effects of an Antibiotic on the Human Gut Microbiota, as Revealed by Deep 16S rRNA Sequencing. *PLOS Biol.* **6**, e280 (2008).

3. Espenberg, M. *et al.* Impact of Reed Canary Grass Cultivation and Mineral Fertilisation on the Microbial Abundance and Genetic Potential for Methane Production in Residual Peat of an Abandoned Peat Extraction Area. *PLOS ONE* **11**, e0163864 (2016).

4. Kandeler, E., Deiglmayr, K., Tscherko, D., Bru, D. & Philippot, L. Abundance of narG, nirS, nirK, and nosZ Genes of Denitrifying Bacteria during Primary Successions of a Glacier Foreland. *Appl. Environ. Microbiol.* **72**, 5957–5962 (2006).

5. Henry, S. *et al.* Quantification of denitrifying bacteria in soils by *nirK* gene targeted real-time PCR. *J. Microbiol. Methods* **59**, 327–335 (2004).

6. Henry, S., Bru, D., Stres, B., Hallet, S. & Philippot, L. Quantitative Detection of the nosZ Gene, Encoding Nitrous Oxide Reductase, and Comparison of the Abundances of 16S rRNA, narG, nirK, and nosZ Genes in Soils. *Appl. Environ. Microbiol.* **72**, 5181–5189 (2006).

7. Jones, C. M., Graf, D. R. H., Bru, D., Philippot, L. & Hallin, S. The unaccounted yet abundant nitrous oxide-reducing microbial community: a potential nitrous oxide sink. *ISME J.* **7**, 417–426 (2013).

8. Rotthauwe, J. H., Witzel, K. P. & Liesack, W. The ammonia monooxygenase structural gene amoA as a functional marker: molecular fine-scale analysis of natural ammonia-oxidizing populations. *Appl. Environ. Microbiol.* **63**, 4704–4712 (1997).

9. Tourna, M., Freitag, T. E., Nicol, G. W. & Prosser, J. I. Growth, activity and temperature responses of ammonia-oxidizing archaea and bacteria in soil microcosms. *Environ. Microbiol.* **10**, 1357–1364 (2008).

10. Wang, M. *et al.* Newly designed primer pair revealed dominant and diverse comammox *amoA* gene in full-scale wastewater treatment plants. *Bioresour. Technol.* **270**, 580–587 (2018).

11. Ueda, T., Suga, Y., Yahiro, N. & Matsuguchi, T. Remarkable N2-fixing bacterial diversity detected in rice roots by molecular evolutionary analysis of nifH gene sequences. *J. Bacteriol.* **177**, 1414–1417 (1995).
